# Preferential rabbit antibody responses to C-termini of NOTCH3 peptide immunogens

**DOI:** 10.1038/s41598-023-36067-7

**Published:** 2023-06-06

**Authors:** Soo Jung Lee, Mitchell B. Gasche, Connor J. Burrows, Akhil Kondepudi, Xiaojie Zhang, Michael M. Wang

**Affiliations:** 1 Department of Neurology, University of Michigan, Ann Arbor, MI 48109 USA; 2 Department of Molecular and Integrative Physiology, University of Michigan, 7725 Medical Science Building II Box 5622, 1137 Catherine St., Ann Arbor, MI 48109-5622 USA; 3 Neurology Service, Department of Veterans Affairs, VA Ann Arbor Healthcare System, Ann Arbor, MI 48105 USA

**Keywords:** Immunology, Applied immunology

## Abstract

Antibodies raised in peptide-immunized rabbits have been used in biological research for decades. Although there has been wide implementation of this approach, specific proteins are occasionally difficult to target for multiple reasons. One consideration that was noted in mice is that humoral responses may preferentially target the carboxyl terminus of the peptide sequence which is not present in the intact protein. To shed light on the frequency of preferential rabbit antibody responses to C-termini of peptide immunogens, we present our experience with generation of rabbit antibodies to human NOTCH3. A total of 23 antibodies were raised against 10 peptide sequences of human NOTCH3. Over 70% (16 of 23) of these polyclonal antibodies were determined to be C-terminal preferring: NOTCH3 peptide-reactive antibodies largely targeted the terminating free carboxyl group of the immunizing peptide. The antibodies that preferred C-terminal epitopes reacted weakly or not at all with recombinant target sequences with extension the C-terminus that eliminated the free carboxyl group of the immunogen structure; furthermore, each of these antisera revealed no antibody reactivity to proteins truncated before the C-terminus of the immunogen. In immunocytochemical applications of these anti-peptide antibodies, we similarly found reactivity to recombinant targets that best binding to cells expressing the free C-terminus of the immunizing sequence. In aggregate, our experience demonstrates a strong propensity for rabbits to mount antibody responses to C-terminal epitopes of NOTCH3-derived peptides which is predicted to limit their use against the native protein. We discuss some potential approaches to overcome this bias that could improve the efficiency of generation of antibodies in this commonly utilized experimental paradigm.

## Introduction

The development and application of research antibodies has been indispensable to the study of proteins. Among research antibodies, anti-peptide antisera have been gained significant favor, largely due to the elucidation of large numbers of protein-coding genetic sequences and to technological advancements in peptide synthesis. These advancements enable antisera to be produced, usually without difficulty, by immunizing animals without the need to produce or purify protein targets^1,2^.

Though largely successful, anti-peptide immunization sometimes fails to generate antibodies useful for detection of protein targets^1,3^. Failures have been attributed to the inability of the peptide antigen to adopt the same conformation as the target protein^4^, burying of the target sequence in inaccessible regions^5^, or to post-translational modification of the target protein that is not reflected in the peptide immunogen.

In a previous study, Liang and colleagues^6^ noted another potential reason for failure to generate usable anti-peptide antibodies: preferential targeting of antibodies to the carboxyl-terminus (C-terminus) of immunizing peptides that is not present in the intact protein. They reported that immunization of mice with an internal epitope of C-CAM1 produced an antibody response largely to the C-terminal end of the immunizing peptide; monoclonal antibodies from the mice reacted to the immunizing peptide in a manner dependent on the C-terminus of the immunogen, but these antibodies did not bind to the intact protein. The investigators concluded that mouse-derived monoclonal antibodies required the carboxylate moiety present in the C-terminus which was eliminated by the peptide bond present in the intact protein.

In another study, Edwards and colleagues^7^ developed a successful approach to generate antibodies against bacterial proteins that relied on immunization of rabbits with small peptides corresponding to the C-termini of individual proteins. This study indicated that rabbits were capable of generating effective responses to C-termini of peptides and that the anti-sera generated to short immunogens were remarkably specific. The relative proportion of antibodies that preferred the C-termini of the peptide immunogens was high for several antibodies reported, as assessed by peptide competition of ELISA assays.

In the current study, we query: (1) the degree to which anti-peptide humoral responses preferentially target the C-terminus of immunizing human peptide sequences in individual rabbits and (2) the frequency of C-terminal preferring humoral responses in a series of rabbits immunized with peptides representing fragments of a human protein. To address these questions, we retrospectively analyzed polyclonal antisera from a series of projects which aimed to generate NOTCH3 antibodies, focusing on the specificity of each antibody preparation for the C-terminus of the peptide used for immunization.

## Results

### NOTCH3 anti-peptide antibodies

**Figure 1:**
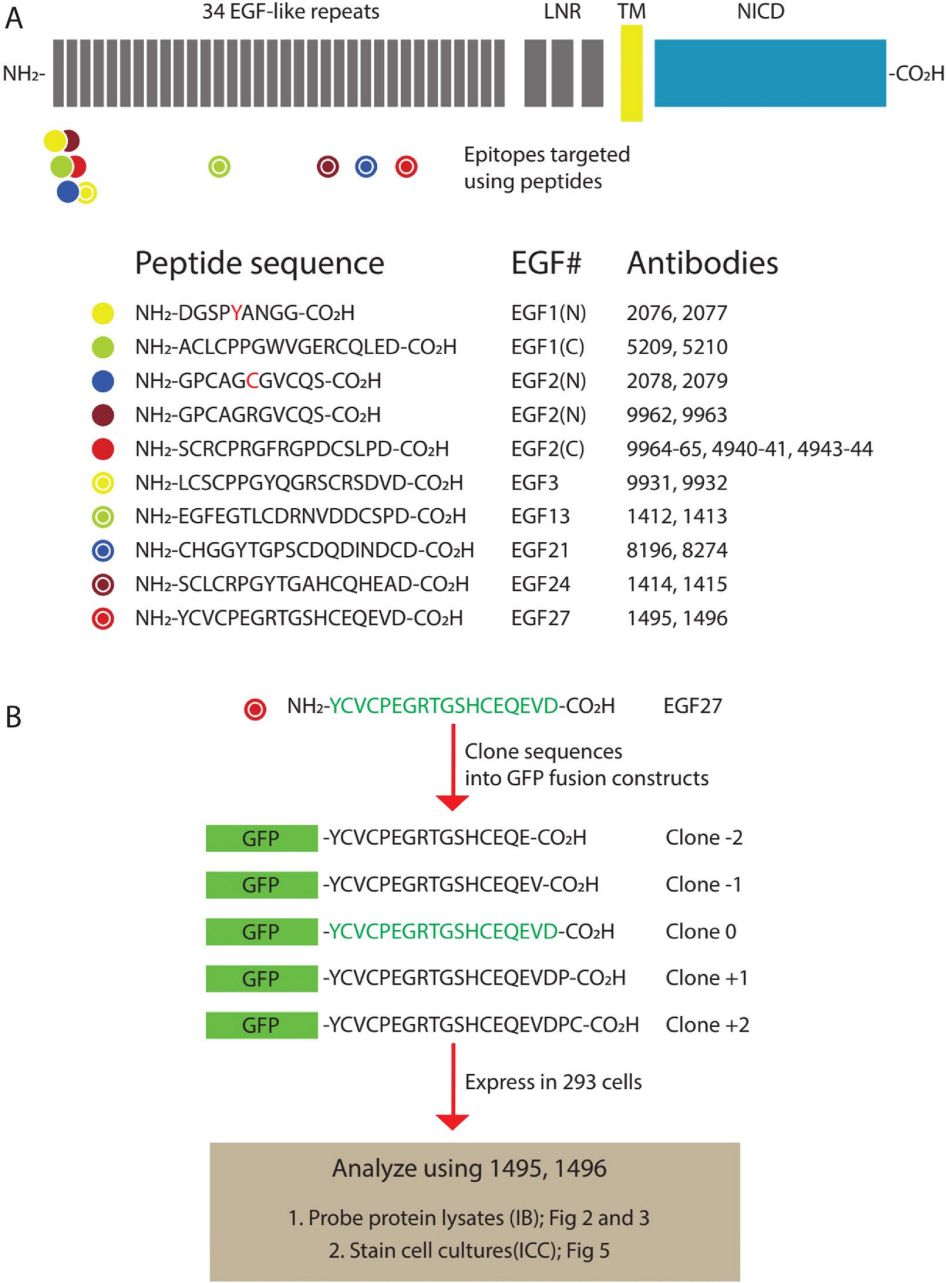
Generation and characterization of anti-peptide antibodies against NOTCH3. (**A**) Positions of NOTCH3 sequences targeted for antibody production and peptide immunogens. Top shows schematic representation of human NOTCH3 protein, composed largely of tandem EGF-like repeats at the N-terminus. Circles depict the positions of peptide sequences used for immunization of rabbits. The corresponding peptide sequences, EGF repeat locations (EGF#), and antibody numbers are displayed below. Wildtype sequences were used, with the exception of two peptides (corresponding to antibodies 2076–2079) that include CADASIL mutant residues (in red). (**B**) Characterization strategy to determine target preferences antisera. Detailed explanation of characterization of antibodies 1495 and 1496 against a peptide sequence from EGF27 exemplifies the approach employed to determine the sequence specificity all antibodies. The immunogen sequence (green) was cloned into the C-terminus of EGFP (GFP) to direct the expression of 5 recombinant variants. Clone 0 terminates precisely at the C-terminus of the immunogen. Clone − 2 is a deletion of two amino acids from Clone 0; Clone − 1 is a deletion of one amino acid from Clone 0; Clone + 1 contains one amino acid extension to Clone 0 (from the wildtype sequence); and Clone + 2 contains two amino acids added to Clone 0). All 5 recombinants and an EGFP clone without any NOTCH3 sequences were expressed in HEK293 cells, and recombinant proteins were analyzed by immunoblotting (IB; see Figs. 2, 3) and by immunocytochemistry (ICC; see Fig. 5) using the corresponding antibodies.

NOTCH3 is a transmembrane receptor (Fig. 1A) that plays multiple roles in development, homeostasis, and pathology. Mutations in NOTCH3 are responsible for the most common cause of inherited stroke and vascular dementia, cerebral autosomal dominant arteriopathy with subcortical infarcts and leukoencephalopathy (CADASIL)^8,9^.

In the course of our work on NOTCH3, we discovered that NOTCH3 is cleaved at two Asp-Pro sequences near the N-terminus which is enhanced in CADASIL^10,11^. These findings required the generation of neo-epitope specific monoclonal antibodies that were specific for C-terminal aspartic acid residues that are generated by Asp-Pro cleavage. These antibodies are specific for sequences with C-terminal aspartic acid; extension of the protein beyond the terminal aspartate residues eliminated binding^10,11^.

We also noted during these studies that the polyclonal sera from immunized rabbits that were used to generate the antibodies against the terminal aspartate sequences were highly enriched for antibodies requiring the C-terminal aspartate; sera contained only small amounts of antibodies capable of reacting to sequences that did not contain the C-terminal aspartate residue. (The data are shown as part of this study.) This prompted us to conduct the current study to determine the frequency of antibody responses that preferentially recognize C-terminal sequences from a large series of independent NOTCH3 antibodies that were prepared by peptide immunization of rabbits.

The regions of NOTCH3 that we have targeted for antibody production are shown in Fig. 1A. The antibodies all target the ectodomain of the protein, the region that is the site of most CADASIL mutations and that is composed of tandem EGF-like repeats^12,13^. EGF-like repeats are enriched in cysteine residues, and all peptides include at least one cysteine residue. The sequences of peptides used are shown in Fig. 1B. Peptides were used to immunize from 2–4 rabbits at a time. Different vendors conducted the experiments^10,11,14^, and immunizations were performed using KLH conjugated peptides whose purity was verified by mass spectroscopy.

Rabbits were immunized in accordance with schedules that were standardized by the vendor contracted to make the antibodies. In all cases, pre-immune sera exhibited minimal reactivity to immunogens and at the end of the immunization schedule, each animal produced serum that was at positive by ELISA at greater than 1:512,000 dilution, indicating a strong humoral response to immunization.

Sera was affinity purified using columns generated by cross-linking peptide to beads. Standard methods were used, including washing with media and elution using acid buffer that was then dialyzed against neutral PBS before the antibody preparations were frozen after stabilization with 20% glycerol. The reactivity of the final polyclonal antibody preparations was verified by ELISA.

### Determination of antigen target range of NOTCH3 antisera

The specificity of each NOTCH3 antibody target sequences was determined by probing immunoblots of recombinant GFP fused to peptide sequences of differing lengths; we illustrate the strategy in an example shown in Fig. 1B. Genetic clones of GFP were fused with DNA sequences encoding the amino acid sequence of each immunizing peptide (called Clone 0 for each antibody). One or two amino acids were deleted from the peptide target to generate Clones − 1 and − 2. In addition, one or two amino acids of the NOTCH3 protein sequence were added to generate Clones + 1 and + 2. An example of GFP clones generated to test sequence targets of antibodies 1495 and 1496 is shown in Fig. 1B. The cDNA expression Clones − 2, − 1, 0, + 1, and + 2 were transfected into cells to generated protein lysates containing fusion GFP proteins and separated on Western blots. The blots were probed with NOTCH3 antisera and GFP antibodies. Subsequently, the ratio of NOTCH3 antibody signal to GFP was taken as a measure of NOTCH3 antibody affinity. If antibodies preferentially react against the C-terminus of the immunizing peptide, one will observe the highest binding signal to the GFP fusion protein from clone 0, with lower signals for the GFP fusion proteins from clones − 2, − 1, + 1, and + 2.

**Figure 2:**
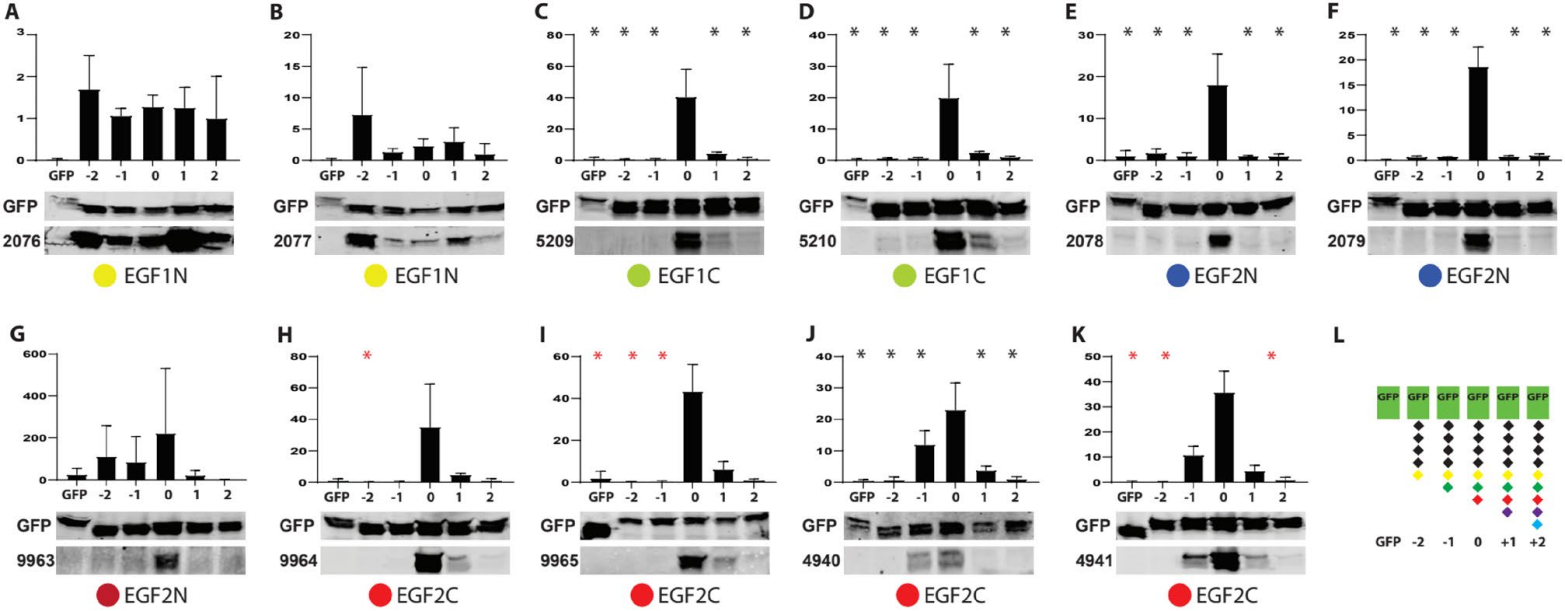
Immunoblot analysis of target preference of NOTCH3 anti-peptide antibodies. Immunoblot analysis of protein lysates of cells transfected with GFP fusions with variable C-terminal residues are shown (**A**–**K**). For each of these blots, HEK293 were transfected with 6 plasmids directing expression of EGFP without NOTCH3 sequences (GFP) or with a complete immunogen sequence (Clone 0) or deletions (− 2 and − 1) or additions (+ 1 or + 2) (see Fig. 1B) that correspond with the antibody listed (see antibody number next to lower blot of each panel). The sequences used for immunization are coded with circles corresponding to Fig. 1A. The blots were probed for both GFP (upper blot) and NOTCH3 antibodies (lower blot) and the ratio of lower/upper signal was determined. All values were normalized to GFP. Two different GFP controls were used that contained different sized non-NOTCH3 C-terminal extensions. (**L**) A schematic representation of proteins produced by transfection showing that for each target sequence, Clone 0 contains the immunogen used (ending in a residue represent by the red diamond), while other clones contain deletions or extensions (purple and blue diamonds). Clone 0 in each panel generates the most signal if an antibody preferentially targets the C-terminus of the immunogen. Immunoblot experiments were performed at least three times for each antibody. Significance with p < 0.05 in comparison with Clone 0 is indicated by a black asterisk (one-way ANOVA for normal data) and a red asterisk (Kruskal–Wallis test for non-parametric data). Full length gels are shown in Supplemental Figs. 3, 4.

**Figure 3:**
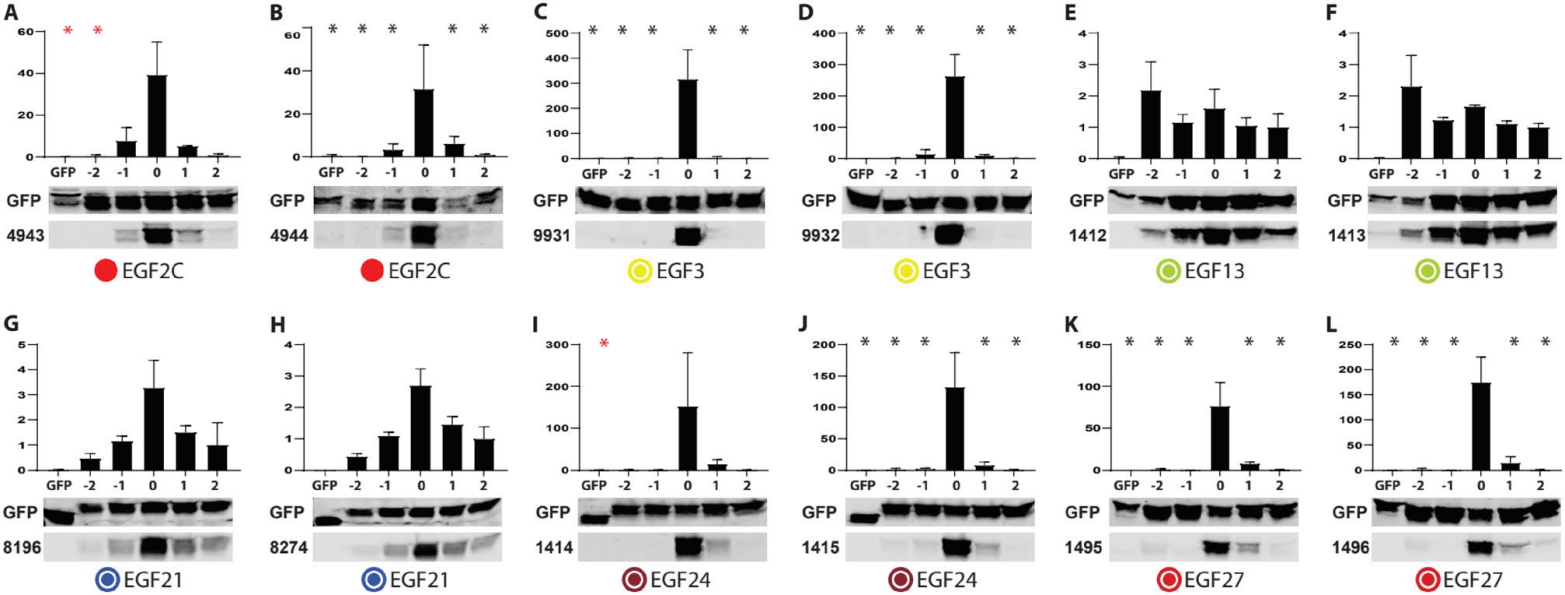
Additional immunoblot analysis of target preference of NOTCH3 anti-peptide antibodies. Please refer to Fig. 2; additional antibodies were evaluated in the same manner in (**A**–**L**). As before, experiments were performed at least three times for each antibody. Full length gels are shown in Supplemental Figs. 3, 4. Significance with p < 0.05 in comparison with Clone 0 is indicated by a black asterisk (one-way ANOVA for normal data) and a red asterisk (Kruskal–Wallis test for non-parametric data).

Immunoblot analysis using NOTCH3 antibodies are shown in Figs. 2, 3 and Supplemental Figs. 1, 2. A large fraction of the antibodies reacted to proteins that contain the carboxyl terminus of the immunizing peptide, with a much lower amount of antibody binding to proteins missing the carboxyl residue (clones − 2 and − 1) and to proteins with amino acid extensions that block the peptide carboxyl residue (clones + 1 and + 2).

Of note, there was very strong concordance of C-terminal specific preference among antibodies that were produced after immunization with a given peptide sequence. Sequences that generated C-terminal preferences did so in 100% of immunized rabbits; in the case of EGF2C, all 6 rabbits qualitatively demonstrated humoral responses that targeted C-terminal epitopes (Figs. 2H–K and 3A,B). Peptides that generated antibodies with attenuated C-terminal preference generated similar preferences in every rabbit tested (for example EGF1N (Fig. 2A,B) and EGF13 (Fig. 3E,F)). The concordance of C-terminal preference is consistent with conservation of humoral responses between individuals that is dependent on the peptide sequence used for immunization.

**Figure 4:**
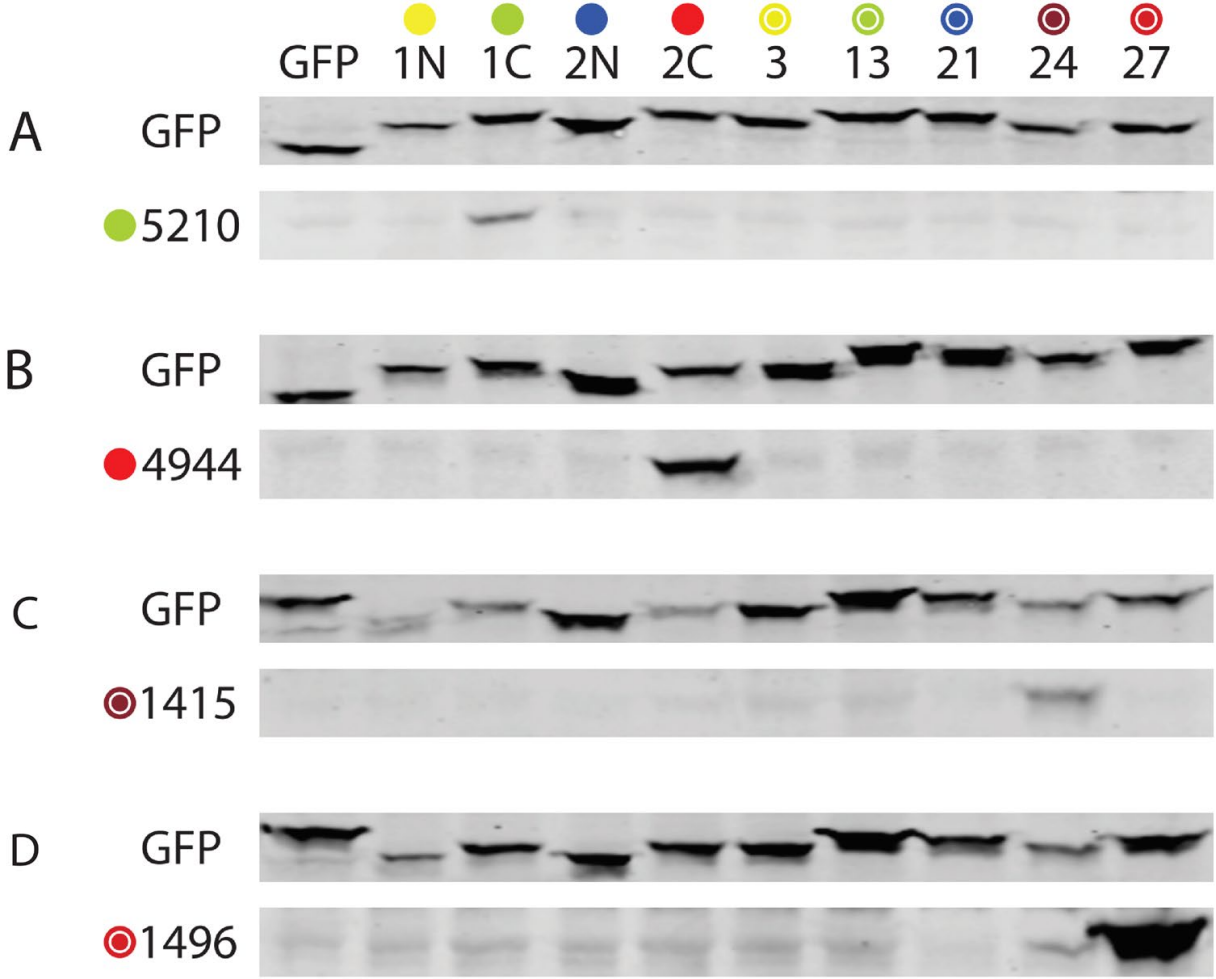
Specificity of antibodies that preferentially target the C-terminus of NOTCH3 peptides. Immunoblots analysis using polyclonal antibodies 5210 (**A**), 4944 (**B**), 1415 (**C**) and 1496 (**D**) was conducted to determine specificity of each antibody against nine independent NOTCH3 sequences. The identity of each protein is shown above the lanes of the blots; lane 1 is a non-NOTCH3 GFP negative control; other lanes represent Clone 0 from the sequences shown in Fig. 1 (markings have been abbreviated for clarity; see Fig. 1A, EGF#). Each panel shows probing for GFP (top blot) compared to probing with the NOTCH3 antibodies (bottom blot of each pair). Full length gels are shown in Supplemental Fig. 5.

A large number of the peptides used to generate antibodies terminated in aspartate residues. In order to verify that antibodies required unique residues besides the terminal aspartate, we performed immunoblotting on all clone 0 proteins that ended in aspartic acid using antibodies directed against C-terminal aspartic acid sequences. Although some antibodies had very faint cross-reactivity against epitopes that were not used for immunization, nine of nine antibodies were most avid for NOTCH3 sequences used for immunization when tested against panels of GFP-NOTCH3 fusions. Figure 4 illustrates four antibodies which were highly specific for the immunizing sequence on immunoblots.

### C-terminal cell staining preference of antibodies for proteins in cell culture

**Figure 5:**
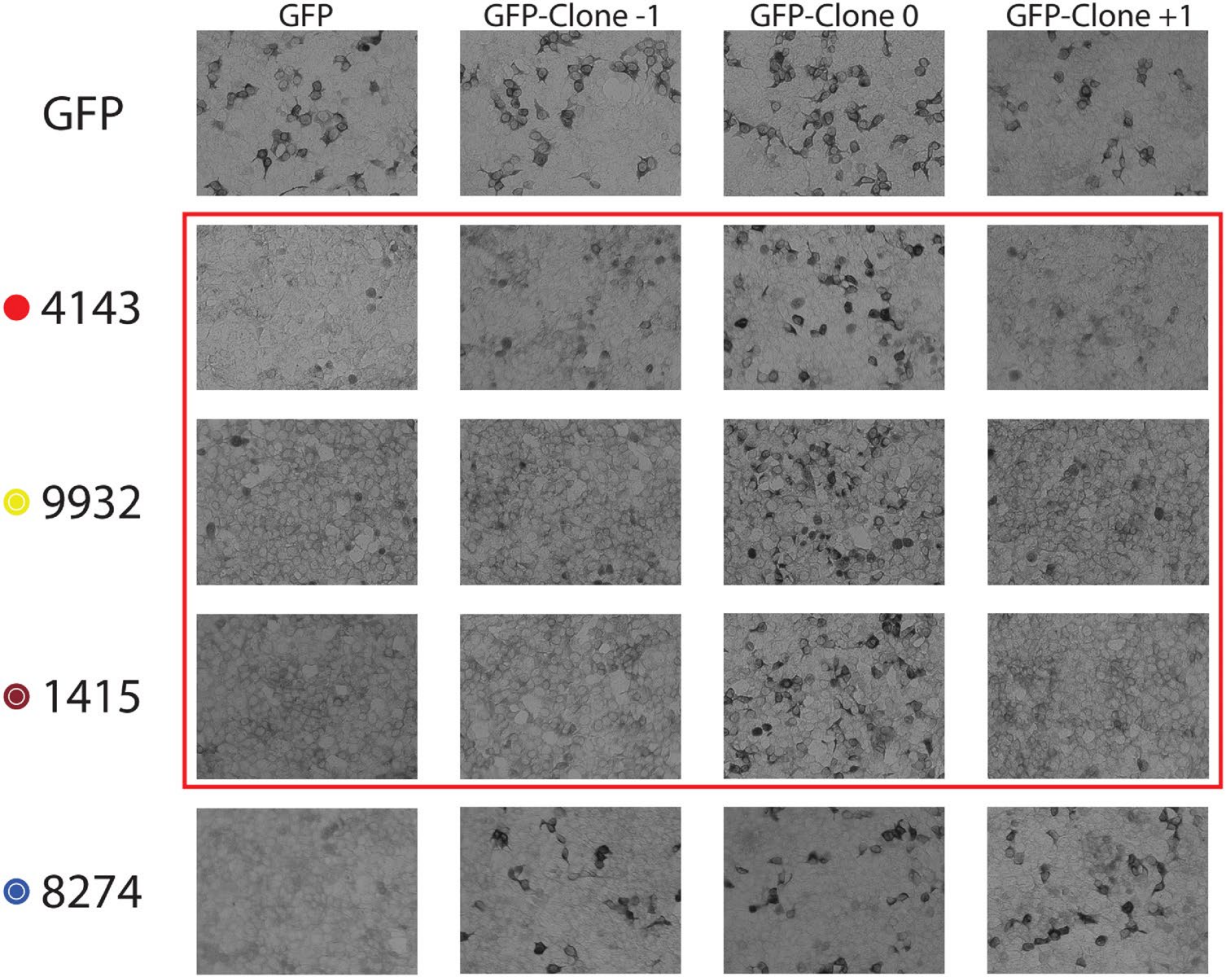
Site preferences of anti-peptide NOTCH3 antibodies by immunocytochemical analysis. HEK293 cells were transiently transfected with recombinant constructs corresponding to top labels. Each set of transfected cells were stained with antibodies on the left. GFP-Clone − 1 corresponds to GFP fused to the corresponding immunizing peptide sequence with one amino acid deletion. GFP-Clone 0 is the GFP fusion to the immunogen sequence (matched at the C-terminus to the peptide sequence in Fig. 1A). GFP-Clone + 1 contains an extra amino acid. All GFP recombinants with NOTCH3 sequences include sequences used for immunization (as shown in Fig. 1A). For example, cells probed with 4143 were transfected with constructs ending in sequences from EGF2(C): SCRCPRGFRGPDCSLP, SCRCPRGFRGPDCSLPD, and SCRCPRGFRGPDCSLPDP.

We also assessed the C-terminal preference of NOTCH3 antibodies by cell staining for proteins expressed in cell culture. Cell cultures were transfected with expression constructs used in Figs. 2, 3. After fixing transfected cells, we performed immunocytochemistry using cognate antibodies. Antibodies with C-terminal preference were expected to show strong staining of cells transfected with clone 0 but not clones − 2, − 1, + 1, or + 2. In general, there was good correspondence between C-terminal preference assessed by immunoblotting and C-terminal preference assessed by immunocytochemistry. For example, in Fig. 5, we show antibody 4143, 9932, and 1415 staining of cells transfected with a series of clones containing its target peptide (clone 0; Fig. 5) and deletions or additions (clones − 1 and + 1; Fig. 5). Cell staining was strongest with Clone 0 and not present above background for Clones − 1 or + 1. This pattern matches the reactivity preferences by immunoblotting There were several antibodies that generated high background staining for both untransfected cells and all transfected groups which made it impossible to assess C-terminal preference. Antibody 8274 showed staining for cells expressing Clones − 1, 0, and + 1 (row 5; Fig. 5) which matched immunoblot results showing that this antibody exhibited non-exclusive C-terminal binding.

## Discussion

Prior work showed that bias towards humoral immune responses in mice to the C-terminus of peptides could limit the diversity and utility of antibodies^6^. Here, we describe the outcomes of a series of 23 rabbit immunizations with 10 different peptides. We demonstrate that a large majority of antisera from these immunizations generate antibodies that reacted primarily to the C-terminal sequences of peptide at the expense of antibodies that are independent of the immunogen’s C-terminus. We also show that the C-terminal epitope preference is similar between individual rabbits and depends on the peptide sequence.

This is the largest study to our knowledge that demonstrates a strong preference for the humoral response in rabbits to target C-termini of human peptide sequences. The results are consistent with Edwards’ study that reported a very effective rate of production of antibodies targeting a class of proteins of interest when C-terminal peptide sequences were used for immunization^7^. Prior polyclonal antibodies raised against peptides ending in aspartate were composed of mostly antibodies that react against C-termini of the peptide immunogens, enabling detection of caspase-generated neo-epitopes^15,16^. It seems likely, based on the collective work, that an approach to generating reagents against mammalian proteins could utilize bias for the immune system of rabbits to generate C-terminal antibodies.

An increase in the number of monoclonal rabbit antibodies, spurred by the availability of rapid workflows that facilitate antibody generation, including B cell cloning technologies, underscore the significance of these observations. The number of clones required to be screened to generated successful antibodies could be high if peptides used for inoculation cause C-terminal specific responses. It is possible that future rabbit monoclonal projects could be increased in efficiency by generating antibodies against the native C-terminus of proteins, as suggested by Edwards et al.^7^.

Can the findings be generalized? One factor that gives us confidence is that we examined multiple peptide sequences as immunogens. Furthermore, the study did not rely on the use of a single facility to produce antibodies; the antibody generation was outsourced to parties that were not involved in the analysis of the sera. In addition, the project was performed over a long time window. Finally, we were able to confirm C-terminal preference of antibodies using two different methods in most cases.

Nevertheless, it is possible that the observed preference for C-termini is overestimated. One limitation of this report is its retrospective nature that analyzed sera that was obtained as part of multiple independent projects not designed to test the hypothesis of this report. The peptides used in this study were all derived from a single extracellular protein. NOTCH3 may be less immunogenic than other proteins because it may be circulating naturally^17^ and because mammalian homologues are well-conserved. In fact, most human peptides used for immunization are nearly identical to rabbit sequences. There are single residue differences in peptides used for antibodies 2076–2079, 9931, 9932, 1412, and 1413 and two amino acids differences for antibodies 1495–1496. The remaining half of the human peptides are identical to rabbit sequences.

As a pragmatic investigation, we did not perform mutational analysis or biochemical modification of peptides that would enable finer details of C-terminal preference. The sequences chosen for this study were not cysteine-free, as NOTCH3 is a cysteine-rich protein. We cannot rule out the possibility that C-terminal preference is driven by the high cysteine content of peptides which may force proteins into specific conformations that bias the humoral response. Future studies with expanded examination of C-terminal titers could be considered, particularly if they are initiated by multiple investigators or industrial scale manufacturers of antibodies that are likely to produce data in a much larger scale than this study. Such a database would enable deeper understanding of specific categories of sequences that are more likely to generate C-terminal reactivity.

Regardless of generalizability, it is clear that C-terminal preference of antibody responses in rabbits occurs; in accordance, decreasing the relative amount of C-terminal antibodies may improve the efficacy of antibody generation. In the future, several methods can be tested to block the C-terminus, including (1) use of cyclic peptides; (2) chemical block of the C-terminus of peptides by esterification; (3) affinity purification using peptides conjugated through the C-terminus or with blocked C-termini; (4) strategies to prevent cysteine residue reactivity which may hide immunogenic regions important for recognition of the middle of peptides; and (5) use of alternative adjuvants or aggressive adjuvant dosing that could help overcome tolerance to conserved epitopes at the core of peptides. It should be noted that Liang et al.^6^ has amidated the C-terminus of C-CAM1 sequences and did not find that it increased the tendency for mice to produce antibodies that recognized the intact target protein; instead, they suggested screening for monoclonal antibodies against a longer C-terminally extended peptide as an immunogen, following by use of a short peptide for screening (or in this case affinity purification). With respect to cysteine alkylation to block cysteine-rich peptides from chemical cross-linking, this could be attempted but its effects have been shown to be attenuated because alkylated cysteines can themselves become the target of antibody responses^18,19^. A natural starting point for testing the effect of adjuvants would be to use alternatives to complete Freunds adjuvant, which was used for all of the animals in this study.

We conclude that C-terminal preference of antibody responses may be a common feature of anti-peptide antibody production; this should be considered as a possible cause of failure to generate antibodies to native protein targets.

## Methods

### Antibodies

Rabbit polyclonal antibodies were generated by commercial vendors using standard procedures which are detailed elsewhere^10,11,14^. All animal experiments were reviewed and approved by the Institutional Animal Care and Use Committees (IACUC) of GenScript and Cocalico Biologics) and performed in accordance with relevant guidelines and regulations, including the recommendations of the Panel on Euthanasia of the American Veterinary Medical Association and ARRIVE guidelines (a control group of non-peptide immunized animals was not included as this is not required for antibody production). Peptide antigens were synthesized corresponding to residues of human NOTCH3 protein shown in Fig. 1 (sequences from Accession: NP_000426.2 GI: 134244285). Antigens were crosslinked to KLH prior to immunization, which was performed with complete Freunds adjuvant for primary immunization and with incomplete Freunds for subsequent boosters. All animals that were injected with peptide antigens described were included in the study. Test bleeds were analyzed by vendors according to their standardized workflow. Serum from terminal bleeds were used for assessment of reactivity profiles in the current study.

### DNA constructs

Double-stranded oligonucleotides encoding target epitopes were inserted into the C-terminus of the EGFP open reading frame by standard ligation procedures with pEGFP-C3 (Clontech); all constructs were sequenced to validate the presence of a continuous open reading frame. As controls, EGFP-C1 or EGFP with an irrelevant C-terminal extension were used to measure background reactivity to the EGFP protein. The use of two negative controls generated different sized products, seen in different immunoblot panels (for example, Fig. 2A–H vs Fig. 2I,K).

### Immunoblotting

We employed methods from previously described work^14,18^. Proteins were resolved on SDS polyacrylamide gels; they were then transferred to nitrocellulose with an iBlot 2 instrument (Invitrogen, method P0 20 V for 1 min/23 V for 4 min/25 V for 2 min); nitrocellulose membranes were blocked using TBST with 5% milk and subsequently probed with primary antibodies in TBST overnight at 4 °C. The primary antibodies were used at a 1:1000 dilution from affinity purified sera. Secondary antibodies in TBST were applied at room temperature for 30 min. Washing after antibody incubations was done three times using TBST at room temperature. Secondary antibody preparations used included donkey anti-mouse IRDye 680RD (Li-Cor #926-68072, 1:10,000 dilution, AB_10953628) and goat anti-rabbit IRDye 800CW (Li-Cor #926-32211, 1:10,000 dilution, AB_2651127). A Li-Cor Odyssey Imager with detection settings at 700 nm and 800 nm and Li-Core Image Studio software was employed for data capture and quantification. Full length immunoblots are displayed in Supplemental Figs. 3–5.

### Cell staining

Cultured HEK293 cells were grown in DMEM and 10% fetal bovine serum. Immunocytochemistry was performed on these cells after they were transfected with EGFP plasmids using PolyJet (SignaGen, cat# SL100688) according to the manufacturer's recommended protocol^20^. After overnight incubation, cells were fixed with formalin. Cells were incubated in blocking solution (2% BSA in PBS) for 30 min and primary antibody (1:200 for GFP antibodies and 1:500 for affinity purified sera) was applied for 2–4 h at room temperature. Biotinylated secondary antibodies in blocking solution at 1:200 dilution were applied for 30 min followed by 15 min incubation of ABC solution prepared according to the manufacturer’s protocol (Vectastain Elite ABC kit, Vector Lab, cat# NC9293436). Finally, DAB incubation for the color reaction was applied for 1–5 min with the ImmPACT DAB HRP Substrate kit (Vector Lab, cat# NC9567138); all washing steps were done with PBS, three to five times; cells were not counterstained. Replicate transfections were also stained for GFP (sc-9996, Santa Cruz Biotechnology).

### Statistics

Data sets were analyzed for normality using the Shapiro–Wilk test. For normally distributed data, comparisons were made using one-way ANOVA with Dunnet’s test for multiple comparisons. For non-parametric analysis, Kruskal–Wallis tests followed by Dunn’s multiple comparison post hoc analysis were used to compare the differences among groups (Prism 8 analysis software). A probability value < 0.05 was regarded as statistically significant.

## Supplementary Information

Supplementary Figures.

## Data Availability

The datasets used and/or analysed during the current study available from the corresponding author on reasonable request.
