# Supplementary material for: Preferential rabbit antibody responses to C-termini of NOTCH3 peptide immunogens
Source: Sci Rep. 2023 Jun 6;13:9156. doi: 10.1038/s41598-023-36067-7 (PMC10244458; doi:10.1038/s41598-023-36067-7)
Supplement: Supplementary file 1 — Supplementary Figures. [file 41598_2023_36067_MOESM1_ESM.pdf]

Supplemental Information:

Preferential rabbit antibody responses to C-termini of NOTCH3 peptide immunogens

Soo Jung Lee[1,3], Mitchell B. Gasche[1],
Connor J. Burrow[1], Akhil Kondepudi[1], Xiaojie Zhang[1,3], and Michael M. Wang[1-3 *]

From the [1]Departments of Neurology, [2]Molecular and Integrative Physiology, University of Michigan, Ann Arbor, MI 48109
[3]Neurology Service, VA Ann Arbor Healthcare System, Department of Veterans Affairs, Ann Arbor, MI 48105

Running Title: Antibody responses to C-termini of NOTCH3 peptides

Address Correspondence to: Michael M. Wang, 7725 Medical Science Building II Box 5622, 1137 Catherine St., Ann Arbor, MI 48109-5622, Tel. 734-763-5453; Fax 734-936-8813; E-Mail: micwang@umich.edu

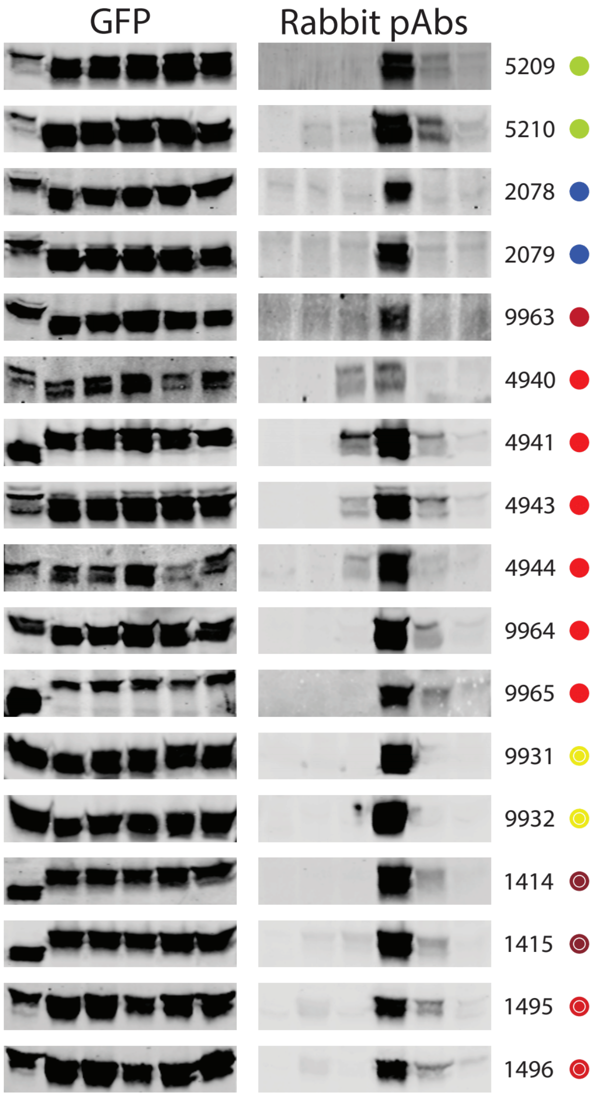

Supplemental Figure 1

Supplemental Figure 1. Polyclonal antisera analysis that demonstrated preference for C-terminus of immunizing peptide. Immunoblots from Fig 2-3 that demonstrate antibody preferences for the C-terminus of immunizing peptides were arrayed together. Proteins were produced by transfection of GFP fusions, as described in Fig 2-3. Antibodies are shown on the right column and described in detail in Fig 1A. See Supplemental Fig 3 for full length gels.

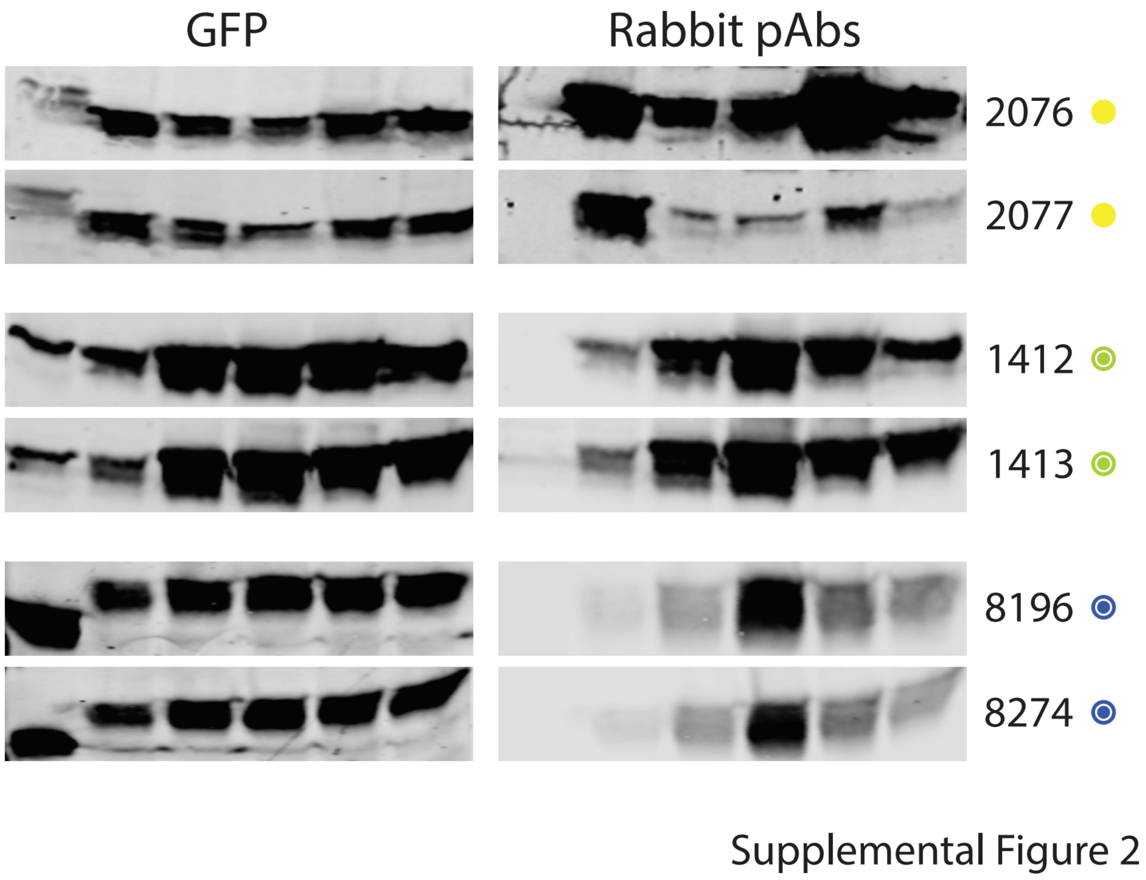

Supplemental Figure 2. Polyclonal antisera analysis that did not demonstrate clear preference for C-terminus of immunizing peptide. Immunoblots from Fig 2-3 that did not demonstrate clear antibody preferences for the C-terminus of immunizing peptides were arrayed together. Proteins were produced by transfection of GFP fusions, as described in Fig 2-3. Antibodies are shown on the right column and described in detail in Fig 1A. See Supplemental Fig 4 for full length gels.

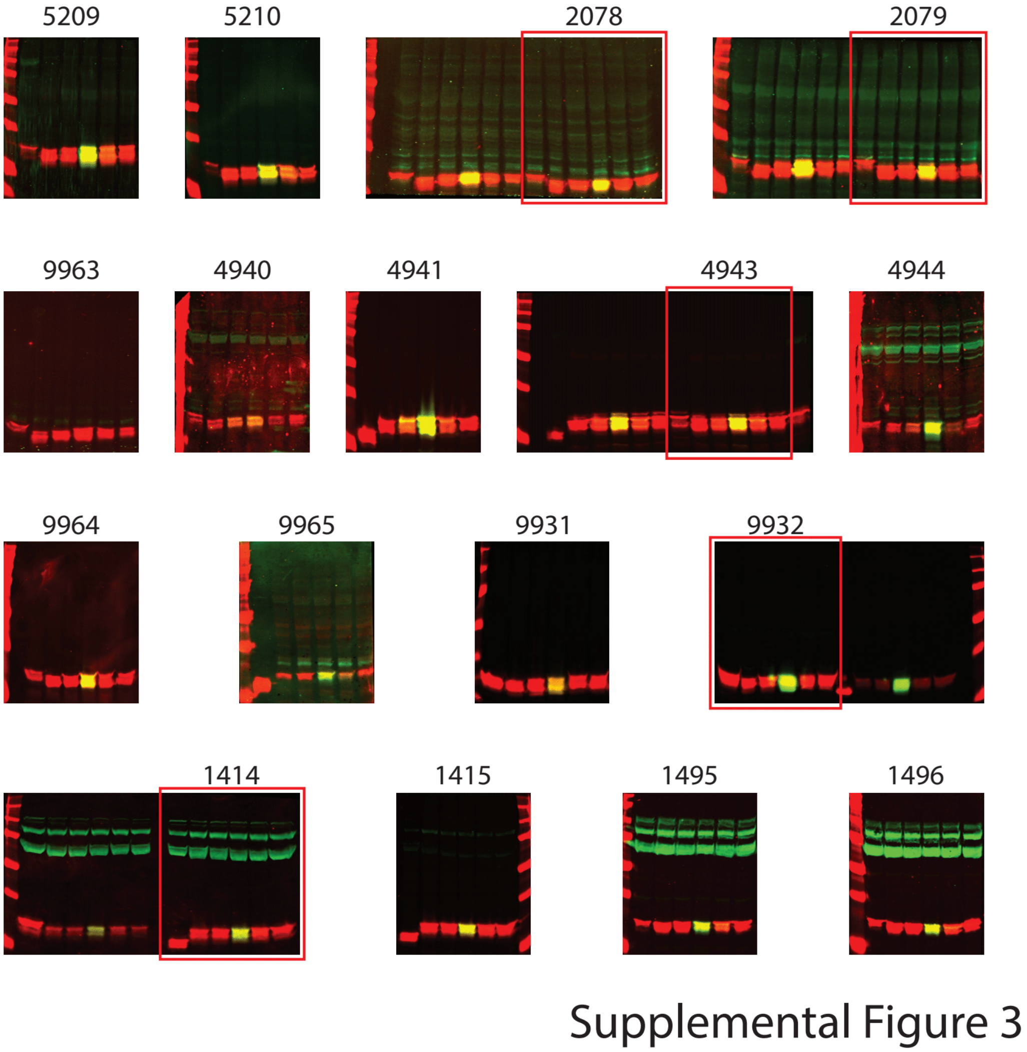

Supplemental Figure 3. Full length gels for polyclonal antisera analysis that demonstrated preference for C-terminus of immunizing peptide. Full length immunoblots analysis in Fig 2-3 and Supplemental Fig 1 are shown. In some cases, two sets of proteins were run on the same gel and probed with the same antibody. The gels used in Fig 2-3 are shown in red boxes.Red corresponds to GFP signal, and green corresponds to polyclonal anti-NOTCH3 signal.

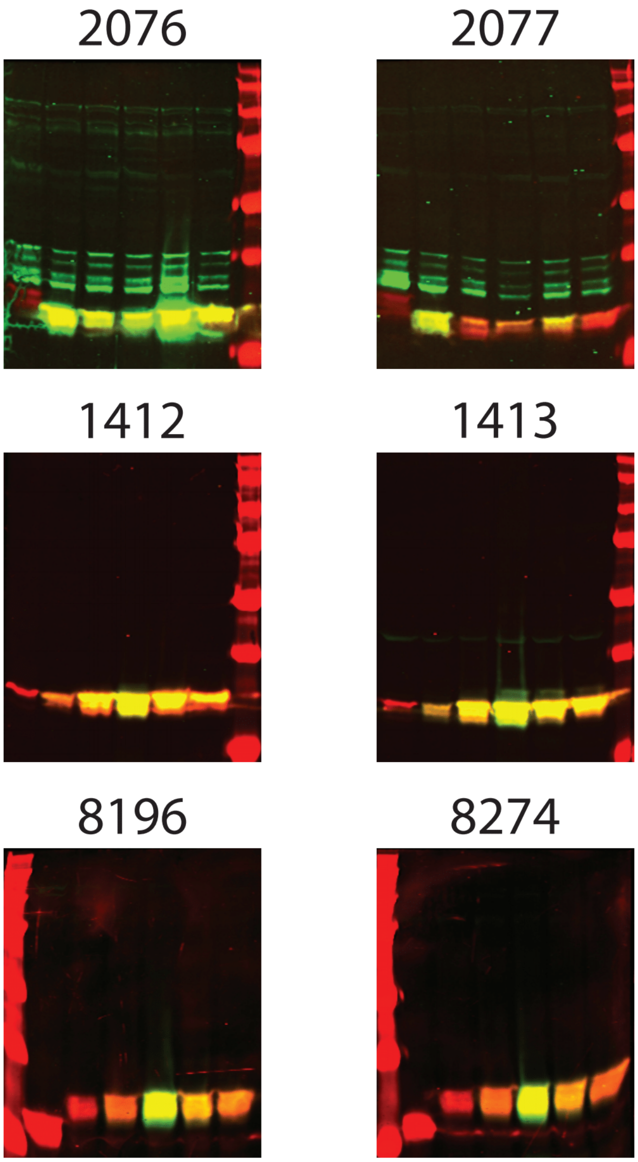

Supplemental Figure 4

Supplemental Figure 4. Full length gels for polyclonal antisera analysis that did not demonstrate preference for C-terminus of immunizing peptide. Full length immunoblots analysis in Fig 2-3 and Supplemental Fig 2 are shown. Red corresponds to GFP signal, and green corresponds to polyclonal anti-NOTCH3 signal.

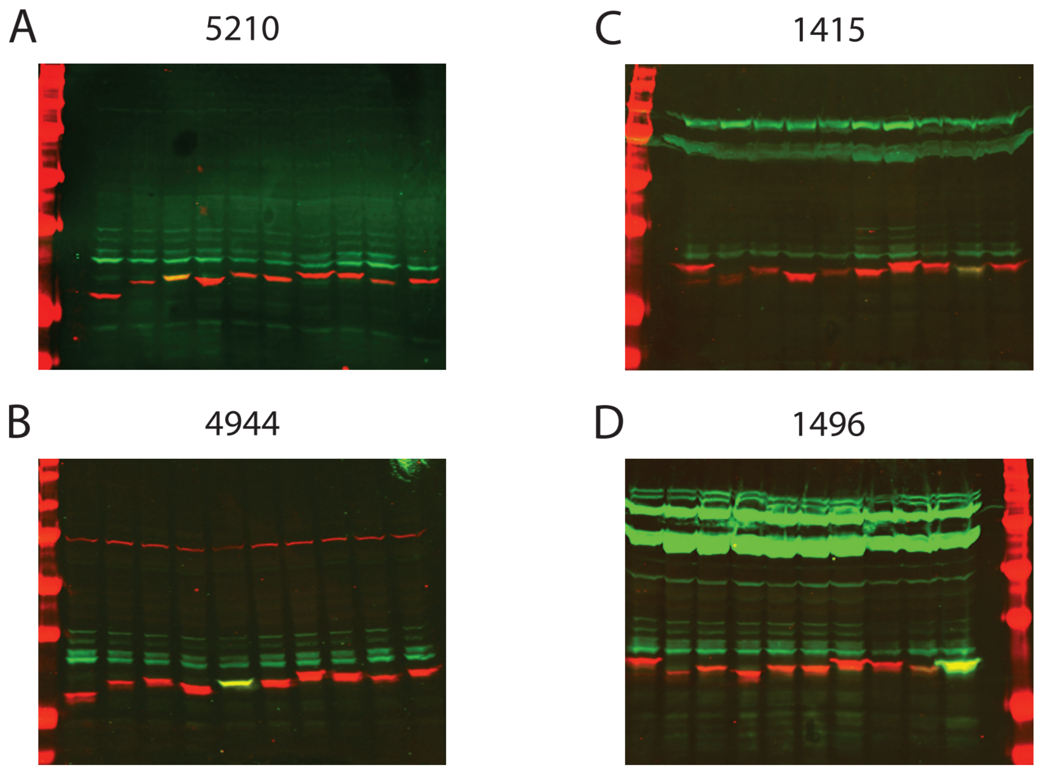

Supplemental Fig 5

Supplemental Figure 5. Full length gels for tests of specificity of polyclonal antisera against individual NOTCH3 sequences. Full length immunoblots analysis in Fig 4 are shown. Red corresponds to GFP signal, and green corresponds to polyclonal anti-NOTCH3 signal.
